# Air pollution impede ALT normalization in chronic hepatitis B patients treated with nucleotide/nucleoside analogues

**DOI:** 10.1097/MD.0000000000034276

**Published:** 2023-10-27

**Authors:** Tyng-Yuan Jang, Chi-Chang Ho, Chih-Da Wu, Chia-Yen Dai, Pau-Chung Chen

**Affiliations:** a Ph.D. Program in Environmental and Occupational Medicine, College of Medicine, Kaohsiung Medical University and National Health Research Institutes, Kaohsiung, Taiwan; b Department of Internal Medicine, Kaohsiung Medical University Hospital, Kaohsiung Medical University, Kaohsiung, Taiwan; c Department of Internal Medicine, Pingtung Hospital, Ministry of Health and Welfare, Ping-Tung, Taiwan; d Institute of Environmental and Occupational Health Sciences, National Taiwan University, Taipei, Taiwan; e Department of Geomatics, National Cheng Kung University, Tainan, Taiwan; f Innovation and Development Center of Sustainable Agriculture, National Chung Hsing University, Tainan, Taiwan; g Department of Public Health, National Taiwan University, Taipei, Taiwan; h Department of Environmental and Occupational Medicine, National Taiwan University Hospital, Taipei, Taiwan; i National Institute of Environmental Health Sciences, National Health Research Institutes, Miaoli, Taiwan.

**Keywords:** air pollution, ALT normalization, HBeAg-negative, HBV, NAs, ozone

## Abstract

Biochemical response is an important prognostic indicator in chronic hepatitis B (CHB) patients receiving nucleotide/nucleoside analogues (NAs). However, the effects of air pollution in alanine aminotransferase (ALT) normalization remain elusive. This longitudinal study recruited 80 hepatitis B e antigen–negative CHB patients who received NAs. ALT levels were measured during the first year of anti-hepatitis B virus therapy. Normal ALT levels were defined as <19 U/L for females and <30 U/L for males, and the risk factors associated with ALT abnormalities were analyzed. The daily estimations of air pollutants (particulate matter ≤2.5 µm in diameter (PM_2.5_), nitrogen dioxide, ozone (O_3_), and benzene) were aggregated into the mean estimation for the previous month based on the date of recruitment (baseline) and 1 year later. Sixteen patients (20.0%) had a baseline ALT > 40 U/L; overall, 41 (51.6%) had an abnormal ALT (≥19 U/L for females and ≥ 30 U/L for males). After 1 year of NA therapy, 75 patients (93.8%) had undetectable hepatitis B virus DNA levels. Mean post-treatment ALT levels were significantly lower than mean pretreatment levels (21.3 vs 30.0 U/L, respectively; *P* < .001). The proportion of patients with a normal ALT was also significantly higher after versus before treatment (71.2% vs 51.2%, respectively; *P* = .001). The strongest factors associated with ALT abnormality after 1 year of NA treatment were body mass index (odds ratio [OR], 1.28; 95% confidence interval [CI], 1.05–1.54; *P *= .01) and ozone level (OR, 1.11; 95% CI, 1.02–1.22; *P *= .02). Among hepatitis B e antigen-negative CHB patients with relatively low viral loads, 1 year of NA treatment improved ALT levels after the adjustment for confounding factors and increased the proportion of patients with normal ALT levels. Air pollution affects the efficacy of ALT normalization.

## 1. Introduction

Hepatitis B virus (HBV) infection is the leading cause of liver cirrhosis and hepatocellular carcinoma (HCC) and poses a great burden on global health. HBV prevalence rates were high prior to universal hepatitis B vaccination.^[1,2]^ Meanwhile, antiviral therapy, including nucleoside/nucleotide analogues (NAs), decreases liver disease progression and reduces the incidence of liver-related complications. The administration of NAs resulted in biochemical remission, decreasing the incidence of hepatic decompensation and HCC.^[3]^ Alanine aminotransferase (ALT) normalization is an important clinical indicator in chronic hepatitis B (CHB) patients receiving NAs. Although the normal upper limit of ALT varies, 40 U/L is conventionally used. However, many studies suggest that a lower ALT has a higher predictive rate of liver diseases.^[4]^ Thus, an ALT <19 U/L for females and <30 U/L for males was suggested by the American Association for the Study of Liver Diseases (AASLD).^[5]^

With the use of novel potent NAs for 1 year, ALT normalization could be achieved in 67% to 78% of patients.^[6–8]^ ALT normalization is affected by virological factors including sex, hepatitis B e antigen (HBeAg) status, HBV DNA level, cirrhosis, and metabolic disorders.^[9]^ Meanwhile, early ALT normalization after NA therapy has been associated with fewer hepatic complications and a lower HCC incidence.^[10]^

Air pollution may cause metabolically associated fatty liver disease (MAFLD), which progresses to liver cirrhosis and HCC.^[11–13]^ Animal studies have suggested that air pollution could trigger oxidative damage and inflammation, which might be involved in the development of chronic liver disease and progression to fibrosis.^[14]^ However, the relationship between air pollution and ALT normalization among CHB patients has not been elucidated. Therefore, here we aimed to address this issue through a longitudinal analysis of a well-characterized CHB cohort receiving NA therapy.

## 2. Methods

### 2.1. Patients

**Figure 1.**
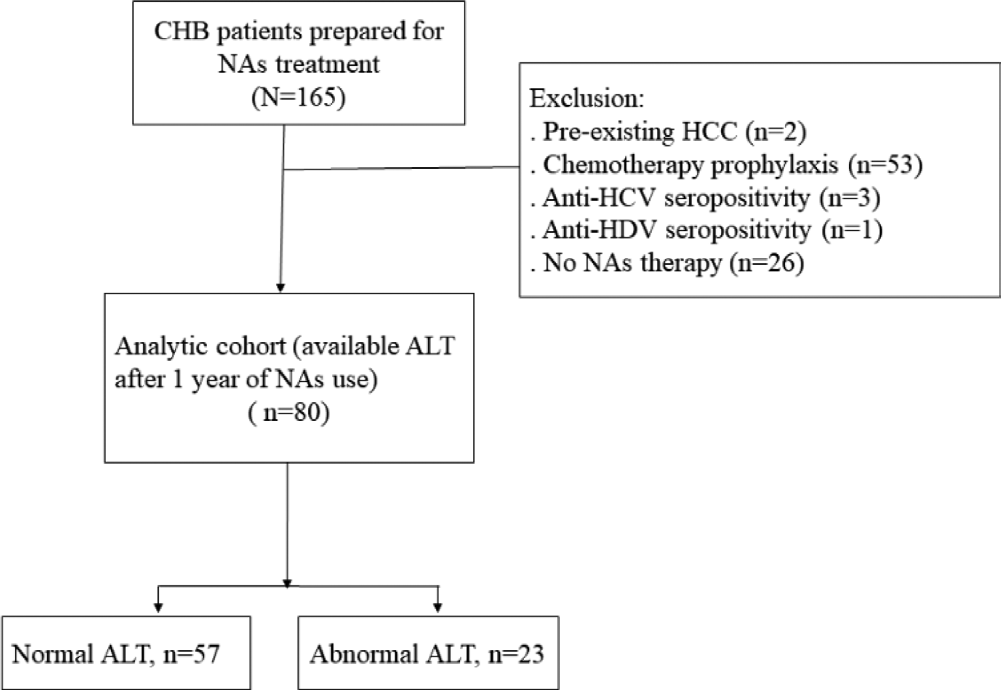
The flowchart of the patient enrollment. ALT = alanine aminotransferase; CHB = chronic hepatitis B; HCC = hepatocellular carcinoma; HCV = hepatitis C virus; HDV = hepatitis D virus; NAs = nucleoside/nucleotide analogue.

Patients with CHB who received NAs were consecutively enrolled in this longitudinal study at a government hospital in Taiwan between 2019 and 2022 (Fig. 1). Treatment indications were based on the regulations of the National Health Insurance Reimbursement of the Ministry of Health and Welfare in Taiwan and/or the patient own expenses. Patients were excluded if they met the following criteria: co-infection with human immunodeficiency virus, hepatitis C virus (HCV), or hepatitis D virus (HDV); were receiving interferon-based therapy; had preexisting HCC or obstructive jaundice; or were using NAs for chemotherapy prophylaxis. This study was conducted in accordance with the Declaration of Helsinki of 1975 as revised in 2008. This study was approved by the ethics committee of Kaohsiung Medical University Hospital.

### 2.2. Laboratory analyses

The biochemical analyses were performed using a multichannel autoanalyzer (Hitachi Inc., Tokyo, Japan). Hepatitis B surface antigen (HBsAg) status was determined using a standard quantitative chemiluminescent microparticle immunoassay (ARCHITECT HBsAg; Abbott Diagnostics, North Chicago, IL, USA). Serum HBV DNA was examined using a standardized automated quantitative polymerase chain reaction assay (COBAS TaqMan HBV test; Roche Diagnostics, Branchburg, NJ; detection limit, 12 IU/mL).^[15]^ HCV antibodies (anti-HCV) were measured using a third-generation enzyme immunoassay (Abbott Laboratories). Anti-HDV antibodies were checked at the time of initiating treatment with anti-HBV agents. Anti-HDV immunoglobulin G (IgG) was screened using an anti-HDV enzyme-linked immunosorbent assay kit (General Biologicals Corporation, Taiwan). Normal ALT levels were defined as <19 U/L in females and <30 U/L in males. Fatty liver was diagnosed by abdominal sonography performed by trained physicians.^[16]^ Liver cirrhosis was diagnosed by the presence of laboratory, radiological, endoscopic, or clinical evidence of portal hypertension and/or cirrhosis.^[17]^ Alcoholism was defined as >20 g/day of alcohol consumption. A low viral load was defined as an HBV DNA level <2000 IU/mL. HCC surveillance was conducted by serum α-fetoprotein protein level determination and induced by vitamin K absence or antagonists-II level measurement and an abdominal ultrasound examination every 3 to 6 months. The protein induced by vitamin K absence or antagonist II reagents were designed for a fully automated chemiluminescence enzyme immunoassay using a LUMIPULSE G1200 system as a leading instrument (Fujirebio Diagnostics).

### 2.3. Air pollution exposure

Individual exposure to air pollutants, such as particulate matter ≤2.5 µm in diameter (PM_2.5_), nitrogen dioxide (NO_2_), ozone (O_3_), and benzene, was evaluated using various hybrid spatial prediction models with daily air pollutant measurements from several Taiwan Air Quality Monitoring (TAQM) stations in Taiwan. Briefly, daily average PM_2.5_ concentrations were measured from approximately 70 TAQM stations from 2006 to 2022. Daily average NO_2_ and O_3_ concentrations were collected from 73 TAQM stations from 2000 to 2022, while average benzene concentrations were measured from 2003 to 2022. The measurements of air pollutants before 2014 were used to develop prediction models to estimate individual air pollutant exposure, and data after 2014 were used to verify model reliability.

Several geospatial and land use datasets, including land use inventory (e.g., residential areas, farmlands, green spaces, point of interest landmark data, and digital road network map), Moderate Resolution Imaging Spectroradiometer, Normalized Difference Vegetation Index, and Digital Terrain Model, were used as predictor variables of air pollutants in the prediction models. Moreover, meteorological data (e.g., daily average temperature and relative humidity) measured by the TAQM stations were also used in some prediction models. All geospatial and land use variables were extracted from different circular buffer ranges surrounding the TAQM stations to represent neighborhood land use conditions.

As for prediction models for air pollutants, a hybrid kriging/land use regression (LUR) model was built to assess the spatiotemporal variation of daily PM_2.5_ concentrations in overall Taiwan. A hybrid kriging/LUR model with a machine learning algorithm (eXtreme Gradient Boosting [XGBoost]) was developed to predict daily NO_2_ concentrations in Taiwan. Daily O_3_ and benzene concentrations were estimated using the LUR model and a LUR with an ensemble machine-learning algorithm (Gradient Boosting, Categorical Boosting, and XGBoost, respectively). The 10-fold cross-validation (CV) methodology with CV-R^2^, adjusted CV-R^2^, and CV root mean square error values were applied to assess the goodness of fit and robustness of the model. Finally, the daily estimations of air pollutants were aggregated into the mean estimation for the previous month based on the date of recruitment (baseline) and 1 year later. Detailed information on the collected air pollutant data, geospatial/land use datasets, and prediction model procedures was provided elsewhere.^[18–20]^

### 2.4. Statistical analyses

Frequencies were analyzed between groups using the chi-squared (χ^2^) test with Yates correction or Fisher exact test. Data are shown as mean ± standard deviation and were compared using analysis of variance, Student *t* test, or the nonparametric Mann–Whitney *U* test. A stepwise logistic regression analysis was applied to analyze factors independently associated with ALT abnormality by analyzing the covariates with values of *P* < .1 in the univariate analysis or factors considered to have potential and clinical relevance. The fibrosis-index 4 (FIB-4) was calculated using the following formula: age (years) × aspartate aminotransferase (AST, U/L)/(platelets in 10^9^/L) × (alanine transaminase [ALT, U/L])^1/2^. Spatial data analysis was performed by using QGIS Desktop 3.0.1 software, and statistical analyses were performed using SPSS 20 (SPSS, Chicago, IL). All statistical analyses were based on 2-sided hypothesis tests, with values of *P* < .05 considered statistically significant.

## 3. Results

### 3.1. Patient characteristics

A total of 80 patients were recruited for the current study (mean age, 63.0 years; 60.0% male). Ten percent of the patients were cirrhotic, and all were HBeAg-negative (Table 1). The mean baseline ALT and FIB-4 levels were 30.0 U/L (range, 9–120 U/L) and 1.9 (range, 0.43–5.31), respectively. Overall, 20.0% of the patients had an ALT level >40 U/L and 51.2% had an abnormal ALT level according to AASLD criteria at the time of enrollment. The NAs included tenofovir alafenamide (50.0%), and entecavir (50.0%).

**Table 1:** Characteristics of the 80 chronic hepatitis B patients preparing for NAs treatment.

	All patients (n = 80)
Age (yr, mean [SD])	63.0 (10.7)
Male, n (%)	48 (60.0)
Diabetes, n/N (%)	11/73 (15.1)
BMI (kg/m^2^, mean [SD])	24.8 (3.5)
AST (U/L, mean [SD])	27.6 (10.7)
ALT (U/L, mean [SD])	30.0 (18.8)
Platelet count (×10^3^ μ/L, mean [SD])	202.9 (60.4)
Total cholesterol (mg/dL, mean [SD])	179.2 (35.3)
TG (mg/dL, mean [SD])	99.7 (50.2)
HDL-C (mg/dL, mean [SD])	57.5 (23.9)
LDL-C (mg/dL, mean [SD])	95.5 (29.8)
FIB-4 (mean [SD])	1.9 (1.0)
AFP (ng/mL, mean [SD])	2.3 (2.0)
PIVKA-II (mAU/mL, mean [SD])	26.7 (11.4)
Cretinine (mg/dL, mean [SD])	0.9 (0.5)
HBeAg seropositivity, n (%)	0 (0)
HBV DNA level (log_10_ IU/mL, mean [SD])	2.5 (1.2)
Fatty liver, n (%)	50 (62.5)
Liver cirrhosis, n (%)	8 (10%)
Alcoholism, n (%)	2 (2.5)
PM_2.5_ (μg/m^3^, mean [SD])	22.2 (11.3)
Ozone (ppb, mean [SD])	38.1 (10.3)
NO_2_ (ppb, mean [SD])	10.3 (5.1)
Benzene (ppbC, mean [SD])	3.4 (5.9)
NAs naïve, n (%)	67 (83.8)
TAF/ETV, n/n	40/40

ALT = alanine aminotransferase; AFP = α-fetoprotein; AST = aspartate aminotransferase; BMI = body mass index; ETV = entecavir; FIB-4 = fibrosis-4 index; HBeAg = hepatitis B e-antigen; HBV = hepatitis B virus; HDL-C, high-density lipoprotein cholesterol; LDL-C, low-density lipoprotein cholesterol; PIVKA-II = protein induced by vitamin K absence or antagonists-II; NAs = nucleoside/nucleotide analogues; NO_2_ = nitrous oxide; PM_2.5_ = particulate matter 2.5; TAF = tenofovir alafenamide; SD = standard deviation; TG = triglycerides.

### 3.2. Changes in serum HBV DNA and ALT levels after 1 year of NA therapy

Post-treatment HBV DNA levels were positive in 6.3% patients. Post-treatment ALT levels were significantly lower than pretreatment levels (21.3 U/L vs 30.0 U/L, respectively; *P* < .001) (Table 2). The proportion of patients with a normal ALT level according to AASLD criteria was also significantly higher after versus before treatment (71.2% vs 48.8%, respectively; *P* = .001) (Supplementary Figure 1, http://links.lww.com/MD/J273). There were no differences in body weight before versus after 1 year of NA therapy.

**Table 2:** Serum parameters compared before and after 1 yr of NAs use.

	Pretreatment level	Post-treatment level	*P* value
AST (U/L, mean [SD])	27.4 (10.7)	23.2 (8.8)	<.001
ALT (U/L, mean [SD])	30.0 (18.8)	21.3 (9.5)	<.001
Normal ALT level*	41 (51.2)	57 (71.2)	.001
Body weight (kilogram, mean [SD])	65.9 (15.8)	66.1 (15.8)	.39
AFP (ng/mL, mean [SD])	2.3 (1.8)	2.1 (1.9)	.08
PIVKA-II (mAU/mL, mean [SD])	25.8 (9.8)	28.6 (10.0)	.04
TG (mg/dL, mean [SD])	102.0 (57.6)	107.7 (50.6)	.51
HDL-C (mg/dL, mean [SD])	56.1 (23.4)	56.0 (21.0)	.93
LDL-C (mg/dL, mean [SD])	95.3 (30.7)	93.0 (25.7)	.58
Total cholesterol (mg/dL, mean [SD])	181.6 (40.5)	168.3 (34.1)	.01
PM_2.5_ (μg/m^3^, mean [SD])	22.6 (11.7)	20.1 (10.2)	.02
Ozone (ppb, mean [SD])	38.1 (10.3)	34.5 (8.7)	.001
NO_2_ (ppb, mean [SD])	10.4 (5.0)	9.8 (4.9)	.24
Benzene (ppbc, mean [SD])	4.7 (8.8)	2.1 (0.9)	.29

AFP = α-fetoprotein; ALT = alanine aminotransferase; AST = aspartate aminotransferase; HDL-C = high-density lipoprotein cholesterol; LDL-C = low-density lipoprotein cholesterol; NO_2_ = nitrous oxide; PIVKA-II = protein induced by vitamin K absence or antagonists-II; PM_2.5_ = particulate matter 2.5; SD = standard deviation; TG = triglycerides.

*< 19 U/L for females and <30 U/L for males.

### 3.3. Factors associated with pretreatment ALT abnormality

Of the 80 CHB patients who prepared to receive NAs, 20.0% had ALT levels >40 U/L and 51.2% patients had abnormal ALT levels according to AASLD criteria after 1 year of NA therapy. Patients with an abnormal ALT level were less likely to be male (46.2% vs 73.2%; *P *= .02) (Table 3).

**Table 3:** Factors affecting pretreatment ALT abnormality.*

	ALT abnormal (n = 39)	ALT normal (n = 41)	*P* value
Age (yr, mean [SD])	63.0 (9.4)	64.1 (10.1)	.98
Male, n (%)	18 (46.2)	30 (73.2)	.02
BMI (kg/m^2^, mean [SD])	25.0 (2.5)	24.7 (4.3)	.68
Diabetes, n (%)	8 (20.5)	6 (14.6)	.56
FIB-4 (mean [SD])	2.0 (1.2)	1.8 (0.9)	.50
AFP (ng/mL, mean [SD])	2.7 (2.2)	2.0 (1.7)	.12
PIVKA-II (mAU/mL, mean [SD])	25.6 (6.9)	27.5 (13.9)	.55
Cretinine (mg/dL, mean [SD])	0.9 (0.4)	1.0 (0.5)	.30
TG (mg/dL, mean [SD])	108.4 (55.4)	91.5 (43.7)	.13
HDL-C (mg/dL, mean (SD))	54.3 (23.7)	60.5 (24.1)	.35
LDL-C (mg/dL, mean [SD])	97.6 (27.1)	93.5 (32.6)	.61
Total cholesterol (mg/dL, mean [SD])	184.6 (33.7)	174.1 (36.5)	.28
Baseline HBV DNA (Log IU/mL, mean [SD])	2.7 (1.4)	2.4 (1.0)	.30
Baseline HBV DNA > 2000 Log IU/mL, n (%)	8 (20.5)	4 (9.8)	.22
Alcoholism, n (%)	1 (2.6)	1 (2.4)	1.00
Fatty liver, n (%)	26 (66.7)	24 (58.5)	.50
Baseline PM_2.5_ (μg/m^3^, mean [SD])	23.8 (11.1)	20.7 (11.5)	.26
Baseline ozone (ppb, mean [SD])	39.3 (10.6)	36.7 (10.0)	.30
Baseline NO_2_ (ppb, mean [SD])	10.9 (5.4)	9.7 (4.8)	.33
Baseline benzene (ppbC, mean [SD])	3.6 (6.2)	3.3 (5.6)	.86
Liver cirrhosis, n (%)	5 (12.8)	3 (7.3)	.48

AFP = α-fetoprotein; ALT = alanine aminotransferase; BMI = body mass index; CI = confidence intervals; ETV = entecavir; FIB-4 = fibrosis-4 index; HBV = hepatitis B virus; HDL-C, high-density lipoprotein cholesterol; LDL-C, low-density lipoprotein cholesterol; NO_2_ = nitrous oxide; OR = odds ratio; PIVKA-II = protein induced by Vitamin K absence or antagonists-II; PM_2.5_ = particulate matter 2.5; SD = standard deviation; TAF = tenofovir alafenamide; TG = triglycerides.

*< 19 U/L for females and <30 U/L for males

### 3.4. Factors associated with ALT abnormality after 1 year of NA therapy

Of the 80 CHB patients who continued receiving NAs for 1 year, 5.0% had an ALT level >40 U/L and 28.8% patients had an abnormal ALT level according to AASLD criteria. Patients with an abnormal ALT had a higher mean body mass index (BMI) (26.8 kg/m^2^ vs 24.1 kg/m^2^; *P *= .004), higher mean O_3_ level (38.4 ppb vs 32.9 ppb; *P *= .02), and a higher proportion of liver cirrhosis (21.7% vs 5.3%; *P *= .02) (Table 4). A logistic regression analysis revealed that the factors associated with ALT abnormalities at 1 year were BMI (odds ratio [OR], 1.28; 95% confidence interval, 1.05–1.54; *P *= .01). and O_3_ level (OR, 1.11; 95% CI, 1.02–1.22; *P *= .02). The best cutoff value of the on-treatment O_3_ level for predicting ALT abnormality was 38.2 ppb (area under the receiver operating characteristic curve, 0.67; *P *= .02) (Supplementary Figure 2, http://links.lww.com/MD/J274). We further used O_3_ > 38 ppb at year 1 as a covariant to analyze the factors affecting persistent ALT abnormalities after 1 year of NA use (Supplementary Table 1, http://links.lww.com/MD/J275). A logistic regression analysis revealed that the factors associated with ALT abnormalities at year 1 were BMI (OR, 1.30; 95% CI, 1.07–1.58; *P *= .01) and O_3_ > 38 ppb at year 1 (OR, 4.08; 95% CI, 1.26–13.22; *P *= .02).

**Table 4:** Factors affecting persistent ALT abnormality* after 1 yr of NAs use.

	ALT abnormality (n = 23)	ALT normalization (n = 57)	*P* value	Logistic regression analysis		
				OR	95% CI	*P* value
Age (yr, mean (SD))	60.4 (12.1)	64.1 (10.1)	.46			
Male, n (%)	13 (56.5)	35 (61.4)	.80			
BMI (kg/m^2^, mean [SD])	26.8 (3.4)	24.1 (3.3)	.004	1.28	1.05–1.54	.01
Diabetes, n (%)	6 (26.1)	8 (14.0)	.21			
FIB-4 (mean [SD])	2.2 (1.4)	1.8 (0.8)	.43			
AFP (ng/mL, mean [SD])	3.2 (2.9)	1.9 (1.4)	.04			
PIVKA-II (mAU/mL, mean [SD])	25.5 (5.1)	27.0 (12.5)	.57			
Cretinine (mg/dL, mean [SD])	0.8 (0.3)	0.9 (0.5)	.51			
TG (mg/dL, mean [SD])	107.4 (49.3)	96.6 (50.6)	.18			
HDL-C (mg/dL, mean [SD])	56.9 (26.9)	57.8 (23.0)	.75			
LDL-C (mg/dL, mean [SD])	88.7 (23.9)	98.2 (31.7)	.24			
Total cholesterol (mg/dL, mean [SD])	175.8 (35.3)	180.6 (35.6)	.39			
Baseline HBV DNA (Log IU/mL, mean [SD])	2.8 (1.2)	2.4 (1.2)	.13			
Baseline HBV DNA > 2000 Log IU/mL, n (%)	4 (17.4)	8 (14.0)	.74			
Detectable HBV DNA at yr-1, n (%)	2 (8.7)	3 (5.3)	.62			
Alcoholism, n (%)	1 (4.3)	1 (1.8)	.50			
Fatty liver, n (%)	18 (78.3)	32 (56.1)	.08			
PM_2.5_ at yr-1 (μg/m^3^, mean [SD])	20.0 (7.5)	20.2 (11.4)	.87			
Ozone at yr-1 (ppb, mean [SD])	38.4 (7.1)	32.9 (7.8)	.02	1.11	1.02–1.22	.02
NO_2_ at yr-1 (ppb, mean [SD])	10.5 (4.8)	9.5 (5.0)	.56			
Benzene at yr-1 (ppbC, mean [SD])	2.4 (0.7)	1.9 (1.0)	.39			
Liver cirrhosis, n (%)	5 (21.7)	3 (5.3)	.04			
ETV/TAF, n/n	9/14	31/26	.32			

AFP = α-fetoprotein; ALT = alanine aminotransferase; BMI = body mass index; CI = confidence intervals; ETV = entecavir; FIB-4 = fibrosis-4 index; HBV = hepatitis B virus; HDL-C, high-density lipoprotein cholesterol; LDL-C, low-density lipoprotein cholesterol; NO_2_ = nitrous oxide; OR = odds ratio; PIVKA-II = protein induced by Vitamin K absence or antagonists-II; PM_2.5_ = particulate matter 2.5; SD = standard deviation; TAF = tenofovir alafenamide; TG = triglycerides.

*< 19 U/L for females and <30 U/L for males.

### 3.5. Factors associated with fatty liver

Of the 80 CHB patients who prepared to receive NAs, 62.5% had fatty liver. Patients with fatty liver had a higher mean BMI (26.4 kg/m^2^ vs 22.3 kg/m^2^; *P *< .001), higher serum triglyceride level (108.5 mg/dL vs 85.1 mg/dL; *P *= .03), and higher proportion of diabetes (26.3% vs 3.3%; *P *= .01) (Table 5). The logistic regression analysis revealed that BMI was associated with fatty liver (OR, 2.41; 95% CI, 1.53–3.80; *P *< .001).

**Table 5:** Factors associated with fatty liver.

	With fatty liver (n = 50)	Without fatty liver (n = 30)	*P* value	Logistic regression analysis		
				OR	95% CI	*P* value
Age (yr, mean [SD])	61.1 (11.1)	66.2 (9.4)	.04			
Male, n (%)	32 (64.0)	16 (53.3))	.36			
BMI (kg/m^2^, mean [SD])	26.4 (2.9)	22.3 (3.0)	<.001	2.41	1.53–3.80	<.001
Diabetes, n (%)	13 (26.0)	1 (3.3)	.01			
FIB-4 (mean [SD])	1.7 (1.0)	2.1 (1.1)	.11			
AFP (ng/mL, mean [SD])	2.4 (2.0)	2.1 (1.9)	.89			
PIVKA-II (mAU/mL, mean [SD])	28.4 (13.9)	25.4 (11.2)	.45			
Cretinine (mg/dL, mean [SD])	0.9 (0.3)	1.0 (0.6)	.22			
TG (mg/dL, mean [SD])	108.5 (54.0)	85.1 (39.8)	.03			
HDL-C (mg/dL, mean [SD])	56.1 (26.0)	60.3 (19.2)	.18			
LDL-C (mg/dL, mean [SD])	94.2 (32.2)	98.1 (25.1)	.49			
Total cholesterol (mg/dL, mean [SD])	176.1 (37.4)	185.3 (30.4)	.27			
Baseline HBV DNA (Log IU/mL, mean [SD])	2.5 (1.2)	2.4 (1.1)	.71			
Alcoholism, n (%)	1 (3.3)	1 (5.2)	1.00			
Baseline PM_2.5_ (μg/m^3^, mean [SD])	24.1 (12.0)	18.8 (9.4)	.14			
Baseline ozone (ppb, mean [SD])	37.9 (11.1)	38.3 (8.9)	.96			
Baseline NO_2_ (ppb, mean [SD])	11.1 (5.5)	8.8 (4.1)	.18			
Baseline benzene (ppbC, mean [SD])	2.2 (0.7)	1.9 (1.2)	.35			
Liver cirrhosis, n (%)	4 (8.0)	4 (13.3)	.47			

AFP = α-fetoprotein; ALT = alanine aminotransferase; BMI = body mass index; CI = confidence intervals; FIB-4 = fibrosis-4 index; HBV = hepatitis B virus; TG, triglycerides; HDL-C, high-density lipoprotein cholesterol; LDL-C, low-density lipoprotein cholesterol; PIVKA-II = protein induced by vitamin K absence or antagonists-II; OR = odds ratio; PM_2.5_ = particulate matter 2.5; NO_2_ = nitrous oxide; SD = standard deviation.

## 4. Discussion

The current study demonstrated that O_3_ impeded ALT normalization in HBeAg-negative patients with a relatively low viral load after 1 year of NA therapy after the adjustment for confounding factors. An O_3_ > 38 ppb at year 1 was associated with a 4-fold possibility of ALT abnormality after NA treatment.

HBV infection causes hepatic inflammation that may lead to liver cirrhosis and HCC. NA therapy improves the biomedical response, reduces fibrosis progression, and decreases the risk of cirrhosis and HCC. The biochemical response is an important surrogate for disease control and prognosis during NA treatment. The causes of abnormal ALT levels include HBV, HCV, and HDV infections,^[21]^ metabolic effects, alcohol use, autoimmune diseases, and drug-related factors. In this study, patients had a relatively low baseline viral load and lower mean ALT level. We excluded other etiologies of severe hepatitis, such as obstructive jaundice, ischemic hepatitis, and autoimmune hepatitis.

The natural history of CHB includes 5 clinical phases.^[22]^ However, there may be a gray zone in which the features do not correspond to any specific phase. The immune-inactive phase is characterized by HBeAg-negative status, a persistently normal ALT level, and a low (<2000 IU/mL) serum HBV DNA level.^[22]^ Without antiviral treatment, the long-term outcome of the immune-inactive phase is good in the absence of advanced fibrosis.^[23]^ However, it is a dynamic phase that can reactivate to an immune-active phase.^[24]^ HBsAg loss affects 1% to 2% of patients per year in the immune-inactive phase.^[25]^ Some studies suggested that peginterferon could increase the HBsAg loss rate, while other studies failed to prove this.^[26,27]^ However, few studies have described NA treatment for these patients. Although increased age might increase the HBsAg loss rate, it also increases the incidence of cirrhosis and hepatic complications.^[28,29]^ Here we demonstrated the short-term benefits of NA treatment, but the long-term outcomes require further investigation.

A viral load of 2000 IU/mL is a strong risk predictor of HCC, an abnormal ALT level, and liver cirrhosis.^[30]^ Some hepatologists suggest NA therapy only if HBV DNA is more than 2000 IU/mL.^[31]^ It has been controversial whether patients need NAs if HBV DNA is less than 2000 IU/mL in non-cirrhotic patients.^[22]^ However, low-level persistent viremia could be associated with liver disease progression, and some patients might transition to the immune-active phase annually.^[32]^ NAs were indicated in patients with advanced fibrosis.

In patients receiving highly potent NAs for 1 year, ALT normalization rates were 67% to 78%.^[8]^ The ALT normalization rates were 95.0% and 71.2%, according to laboratory (ALT >40 U/L) and AASLD criteria after 1 year of NA treatment, respectively. ALT normalization after NA therapy was an important indicator for long-term prognosis.^[10,33]^ Wong et al reported that in CHB patients who received NA treatment, the risk of hepatic complications was significantly lower in patients who had ALT normalization during NA treatment than in those with abnormal ALT.^[10]^ We also had reported that NAs improved the ALT levels in non-cirrhotic, HBeAg-negative patients with low viral load after 1 year of therapy by adjusting for confounding factors.^[34]^ Moreover, on-treatment ALT levels were found to have a dose-dependent effect on the risk of HCC.^[10,33]^

In this study, we adapted the strict AASLD criteria for ALT normalization analysis, which might explain why there were no differences between baseline ALT abnormalities and phenotypes such as HBV DNA, fatty liver, liver cirrhosis, and air pollution. However, some patients still did not normalize ALT after 1 year of NAs use, and higher BMI and O_3_ were the main reasons. These 2 factors might affect ALT normalization, even under strict AASLD criteria. Metabolic disorders, such as diabetes and higher BMI, have been reported as risk factors for ALT abnormalities.^[21,35,36]^ Some studies suggest body weight gain after NA treatment.^[37]^ In the study, there were no differences in body weight before and after 1 year of NA therapy, and were not a factor affecting ALT normalization.

Air pollution may cause abnormal ALT levels; however, its role in CHB patients was unknown.^[38]^Air pollution can cause MAFLD.^[13]^ Air pollution may trigger oxidative damage and inflammation, which might be involved in the development of chronic liver disease.^[14]^ Mice studies suggested that air pollution could activate Kupffer cells and produce of cytokines through activating endoplasmic reticulum stress responses, and promoting collagen deposition and progression to fibrosis. Air pollution has also been reported to be related to liver cirrhosis and HCC.^[11,12]^ However, the relationship between air pollution and ALT normalization among CHB patients was not been elucidated. O_3_, an air pollutant, was reported to cause abnormal ALT levels.^[38]^ In this study, we found that O_3_ might impede ALT normalization after NA treatment, but the mechanism and long-term outcomes should be further studied.

This study had some limitations. First, we enrolled only a small number of patients and performed a short-term follow-up. Second, the air pollution level was calculated as the mean value for the previous 1 month before the ALT level was examined. We did not compare the effects of different intervals between the 2 variables. Third, we recorded the home address by postcode; however, we did not record the area of activity throughout the day. Moreover, that study lacked information on MAFLD, and we did not evaluate the potential influence of MAFLD on ALT normalization. Nevertheless, we considered potential confounding factors, such as BMI, diabetes, and fatty liver, on ultrasonography. TAF therapy was reported elevating total cholesterol and triglycerides in a previous study.^[39]^ The current study had small samples and was not able to echo the previous study (Supplementary Table 2, http://links.lww.com/MD/J276). Although the participants were enrolled at a medium-sized hospital, CHB surveillance continues. To the best of our knowledge, this is the first report on air pollution impeding ALT normalization in patients with CHB.

In conclusion, 1 year of NA treatment improved ALT levels in HBeAg-negative patients with a relatively low viral load. A higher BMI and O_3_ level may impede ALT normalization. Further studies are required to determine the long-term outcomes of these patients.

## Acknowledgments

This work was supported in part by a grant from 1. Kaohsiung Medical University (NSTC 112-2923-B-037 -002 -MY3, NSTC 112-2314-B-037 -076 -MY3) 2. KMUH109-9R077.

## Author contributions

**Data curation:** Chi-Chang Ho.

**Methodology:** Chih-Da Wu.

**Writing – original draft:** Tyng Yuan Jang.

**Writing – review & editing:** Chia-Yen Dai, Pau-Chung Chen.

## Supplementary Material








