# Supplementary material for: Air pollution impede ALT normalization in chronic hepatitis B patients treated with nucleotide/nucleoside analogues
Source: Medicine (Baltimore). 2023 Oct 27;102(43):e34276. doi: 10.1097/MD.0000000000034276 (PMC10615411; doi:10.1097/MD.0000000000034276)

Supplementary Figure 1

Pre-treatment and post-treatment ALT level

Normal ALT was defined as <19 U/L for females and <30 U/L for males.

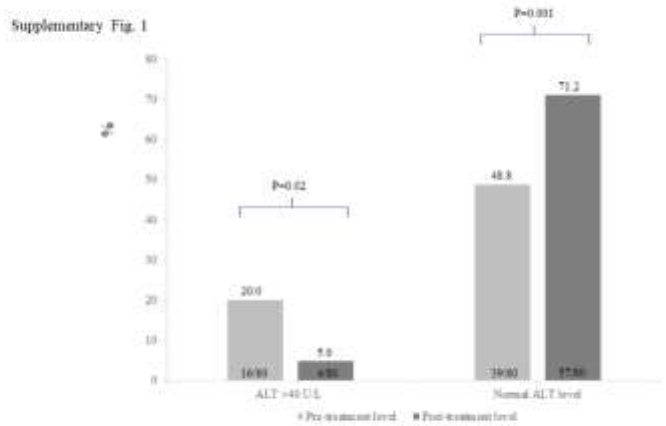

Supplement: Supplementary file 1 [file medi-102-e34276-s001.pdf]
