# Supplementary material for: Air pollution impede ALT normalization in chronic hepatitis B patients treated with nucleotide/nucleoside analogues
Source: Medicine (Baltimore). 2023 Oct 27;102(43):e34276. doi: 10.1097/MD.0000000000034276 (PMC10615411; doi:10.1097/MD.0000000000034276)

Supplementary Figure 2

ROC curve of the on-treatment O_3 level for predicting ALT abnormality (area under the ROC, 0.67; *P* = 0.02)

ROC: Receiver operating characteristic curve

Supplementary Fig. 2

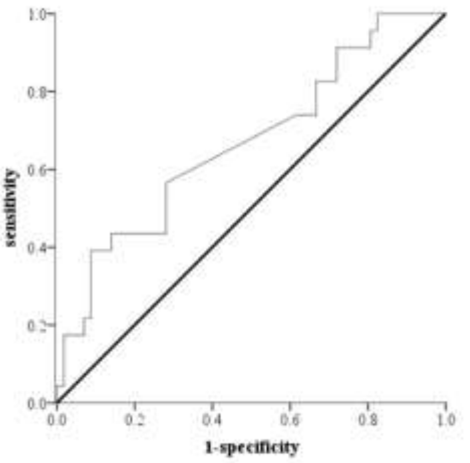

Supplement: Supplementary file 2 [file medi-102-e34276-s002.pdf]
