# Supplementary material for: Air pollution impede ALT normalization in chronic hepatitis B patients treated with nucleotide/nucleoside analogues
Source: Medicine (Baltimore). 2023 Oct 27;102(43):e34276. doi: 10.1097/MD.0000000000034276 (PMC10615411; doi:10.1097/MD.0000000000034276)
Supplement: Supplementary file 3 [file medi-102-e34276-s003.pdf]

Supplementary Table 1. Factors affecting persistent ALT abnormality† after 1 year of NAs use

	ALT abnormality (n=23)	ALT normalization (n=57)	P value	Logistic regression analysis		
				**OR**	**95% C.I.**	**P value**
Age (years, mean (SD))	60.4 (12.1)	64.1 (10.1)	0.46			
Male, n (%)	13 (56.5)	35 (61.4)	0.80			
BMI (kg/m^2, mean (SD))	26.8 (3.4)	24.1 (3.3)	0.004	1.30	1.07-1.58	0.01
Diabetes, n (%)	6 (26.1)	8 (14.0)	0.21			
FIB-4 (mean (SD))	2.2 (1.4)	1.8 (0.8)	0.43			
AFP (ng/mL, mean (SD))	3.2 (2.9)	1.9 (1.4)	0.04			
PIVKA-II (mAU/mL, mean (SD))	25.5 (5.1)	27.0 (12.5)	0.57			
Cretinine (mg/dL, mean (SD))	0.8 (0.3)	0.9 (0.5)	0.51			
TG (mg/dL, mean (SD))	107.4 (49.3)	96.6 (50.6)	0.18			
HDL-C (mg/dL, mean (SD))	56.9 (26.9)	57.8 (23.0)	0.75			
LDL-C (mg/dL, mean (SD))	88.7 (23.9)	98.2 (31.7)	0.24			
Total cholesterol (mg/dL, mean (SD))	175.8 (35.3)	180.6 (35.6)	0.39			
Baseline HBV DNA (Log IU/mL, mean (SD))	2.8 (1.2)	2.4 (1.2)	0.13			
Baseline HBV DNA >2000 Log IU/mL, n (%)	4 (17.4)	8 (14.0)	0.74			
Detectable HBV DNA at year 1, n (%)	2 (8.7)	3 (5.3)	0.62			
Alcoholism, n (%)	1 (4.3)	1 (1.8)	0.50			
Fatty liver, n (%)	18 (78.3)	32 (56.1)	0.08			
$PM_{2.5}$ at year-1 (ug/m^3, mean (SD))	20.0 (7.5)	20.2 (11.4)	0.87			
Ozone >38.0 ppb at year-1, n (%)	10 (43.5)	9 (15.8)	0.02	4.08	1.26-13.22	0.02

NO_2 at year-1 (ppb, mean (SD))	10.5 (4.8)	9.5 (5.0)	0.56
Benzene at year-1 (ppbC, mean (SD))	2.4 (0.7)	1.9 (1.0)	0.39
Liver cirrhosis, n (%)	5 (21.7)	3 (5.3)	0.04
ETV/TAF, n/n	9/14	31/26	0.32

Note: SD: standard deviation; ALT: alanine aminotransferase; BMI: body mass index; HBV: hepatitis B virus; TG, triglycerides; HDL-C, high-density lipoprotein cholesterol; LDL-C, low-density lipoprotein cholesterol; AFP: α-fetoprotein; PIVKA-II: Protein induced by Vitamin K absence or antagonists-II; FIB-4: fibrosis-4 index;; OR: odds ratio; CI: confidence intervals; $PM_{2.5}$: particulate matter 2.5; NO_2: Nitrous oxide ; ETV: entecavir; TAF: tenofovir alafenamide [†]<19 U/L for females and <30 U/L for males
