# Supplementary material for: Air pollution impede ALT normalization in chronic hepatitis B patients treated with nucleotide/nucleoside analogues
Source: Medicine (Baltimore). 2023 Oct 27;102(43):e34276. doi: 10.1097/MD.0000000000034276 (PMC10615411; doi:10.1097/MD.0000000000034276)
Supplement: Supplementary file 4 [file medi-102-e34276-s004.pdf]

Supplementary Table 2. Serum parameters compared before and after 1 year of TAFs use

	**Pre-treatment level**	**Post-treatment level**	**P value**
AST (U/L, mean (SD))	26.9 (10.4)	24.1 (8.7)	0.01
ALT (U/L, mean (SD))	27.4 (14.5)	22.2 (10.3)	0.003
Body weight (kilogram, mean (SD))	62.4 (15.2)	62.8 (15.5)	0.21
AFP (ng/mL, mean (SD))	2.2 (2.2)	2.2 (1.9)	0.78
PIVKA-II (mAU/mL, mean (SD))	25.5 (11.3)	31.3 (10.1)	0.002
TG (mg/dL, mean (SD))	105.3 (57.6)	114.6 (57.2)	0.50
HDL-C (mg/dL, mean (SD))	63.3 (30.1)	60.2 (27.7)	0.24
LDL-C (mg/dL, mean (SD))	80.7 (21.9)	83.3 (25.5)	0.35
Total cholesterol (mg/dL, mean (SD))	173.7 (41.8)	164.4 (38.4)	0.15
$PM_{2.5}$ (ug/m^3, mean (SD))	20.7 (9.3)	18.0 (8.7)	0.04
Ozone (ppb, mean (SD))	39.2 (9.3)	35.3 (6.2)	0.03
NO_2 (ppb, mean (SD))	10.5 (4.2)	9.9 (4.7)	0.15
Benzene (ppbc, mean (SD))	2.6 (0.7)	2.1 (0.8)	0.12

Note: SD: standard deviation; TAF: tenofovir alafenamide; AST: aspartate aminotransferase; ALT: alanine aminotransferase; TG, triglycerides; HDL-C, high-density lipoprotein cholesterol; LDL-C, low-density lipoprotein cholesterol; AFP: α-fetoprotein; PIVKA-II: Protein induced by Vitamin K absence or antagonists-II; $PM_{2.5}$: particulate matter 2.5; NO_2: Nitrous oxide [†]<19 U/L for females and <30 U/L for males
